# Estimated Uncovered Costs For HIV Preexposure Prophylaxis In The US, 2018

**DOI:** 10.1377/hlthaff.2022.00523

**Published:** 2023-04

**Authors:** Robert A. Bonacci, Michelle Van Handel, Rebecca Huggins, Seidu Inusah, Dawn K. Smith

**Affiliations:** Centers for Disease Control and Prevention, Atlanta, Georgia.; Centers for Disease Control and Prevention.; Centers for Disease Control and Prevention.; Centers for Disease Control and Prevention.; Centers for Disease Control and Prevention.

## Abstract

The cost of HIV preexposure prophylaxis (PrEP) medication and care is a key barrier to PrEP use. Using population-based surveys and published information, we estimated the number of people with uncovered costs for PrEP care among US adults with PrEP indications, stratified by HIV transmission risk group, insurance status, and income. Accounting for existing PrEP payer mechanisms, we estimated annual uncovered costs for PrEP medication, clinical visits, and laboratory testing based on the 2021 PrEP clinical practice guideline. Of 1.2 million US adults with PrEP indications in 2018, we estimated that 49,860 (4 percent) of them had PrEP-related uncovered costs, including 32,350 men who have sex with men, 7,600 heterosexual women, 5,070 heterosexual men, and 4,840 people who inject drugs. Of those 49,860 people with uncovered costs, 3,160 (6 percent) incurred $18.9 million in uncovered costs for PrEP medication, clinical visits, and lab testing, and 46,700 (94 percent) incurred $83.5 million in uncovered costs for only clinical visits and lab testing. The total annual uncovered costs for adults with PrEP indications were $102.4 million in 2018. The proportion of people with uncovered costs for PrEP is less than 5 percent among adults with PrEP indications, but the magnitude of costs is significant.

As of 2018 an estimated 1.2 million people in the US had indications for HIV preexposure prophylaxis (PrEP).^1^ PrEP is highly effective in preventing acquisition of HIV and is indicated for men and women with sexual or injection drug behaviors that increase their chances of getting HIV.^2^ PrEP is a critical component of the Ending the HIV Epidemic in the US (EHE) initiative’s “prevent” pillar, which has set a goal for PrEP to be prescribed to at least 50 percent of people with indications for PrEP by 2025.^3^

However, only 30 percent of people with PrEP indications received a PrEP prescription in 2021.^4^ Barriers to effective PrEP use are wide ranging and span the PrEP continuum, from lack of awareness of PrEP to limited opportunities for PrEP uptake and low retention in care.^5,6^ The cost of PrEP medication, clinical visits, and laboratory testing (hereafter collectively referred to as PrEP medication and clinical care) and limited access to health insurance have been identified frequently as barriers to PrEP use.^5,7–10^ A 2015 analysis by Dawn Smith and colleagues^11^ estimated that approximately 90,000 people in the US (approximately 8 percent of people with PrEP indications) had uncovered costs for PrEP medication and clinical care in 2015, suggesting that a substantial number of people have financial barriers.

Since that study, the US health insurance and PrEP coverage landscapes have changed significantly, affecting out-of-pocket spending for PrEP medication and clinical care. In 2019 the US Preventive Services Task Force issued a grade A recommendation for oral PrEP. As a result, beginning January 1, 2021, under the Affordable Care Act (ACA), Medicaid expansion programs and most private insurance plans (not including grandfathered plans or short-term limited-duration plans) are required to cover PrEP without cost sharing.^12^ As of September 2022, thirty-nine US states (plus Washington, D.C.) had expanded Medicaid eligibility under the ACA.^13^ In addition, the Labor, Health and Human Services, and Treasury departments issued a frequently asked questions document in July 2021 clarifying that all plans and issuers not exempted under the ACA must cover PrEP, consistent with the US Preventive Services Task Force recommendation, including clinical visits and laboratory tests related to the evaluation for and monitoring of PrEP, without cost sharing.^14^ Some state PrEP assistance programs and the federal government’s Ready, Set, PrEP Program, initiated in late 2019, were launched during this time as well.^15,16^ Collectively, these developments represent a significant expansion of insurance coverage and financial assistance programs for people initiating or continuing PrEP use. In addition, the introduction in 2021 of multiple manufacturers of generic tenofovir disoproxil fumarate/emtricitabine (TDF/FTC), the primary PrEP medication and the equivalent of brand-name Truvada, has led to large declines in medication price.^17^

Given these recent changes to the PrEP payment landscape, we sought to update estimates of the number of people in the US with uncovered costs for PrEP medication and clinical care and the total annual uncovered costs under 2021 guideline and coverage conditions. This information can inform ongoing policy discussions regarding the establishment of a national PrEP assistance program.

## Study Data And Methods

### Estimating Uncovered Costs For PrEP

Online appendix exhibit 1^18^ illustrates the framework guiding this analysis to identify people with uncovered costs for PrEP medications and clinical care. Using a similar framework and methods, we updated the prior analysis by Smith and colleagues,^11^ incorporating recent changes to the PrEP coverage landscape. We considered uncovered costs to represent the total amount that people would pay for PrEP medication and care beyond any insurance coverage or manufacturer’s medication assistance program. Considering the current PrEP coverage landscape, PrEP medication and clinical care are covered without cost sharing by nonexempt private insurance plans and Medicaid expansion programs. Medicare and traditional Medicaid programs cover PrEP medication and clinical care but may require cost sharing. For people who lack insurance or who are subject to cost sharing, state, jurisdictional, or manufacturer medication assistance programs may cover costs related to PrEP medication or clinical care (scope of coverage varies by program).We did not have the data necessary to incorporate cost sharing for people with public or exempted private insurance in this framework; thus, people with those plans were included in the general private and public insurance groups.

For publicly and privately insured people, we estimated that 1 percent of those with insurance coverage would have a PrEP prescription denied by their insurance, whether from coverage policies not consistent with federal and state regulations or from administrative errors, and that this did not vary by risk group.^11^ For uninsured people, we estimated that 13 percent were ineligible because of immigration status and that 8 percent fell into the Medicaid coverage gap, which includes people in states that have not expanded Medicaid who have incomes above their state Medicaid program’s eligibility threshold but below the threshold for ACA Marketplace subsidies.^19^ We considered all of the above people to have uncovered costs for PrEP medication and clinical care. We also estimated the number of uninsured people who neither had immigration status restrictions nor were in the Medicaid coverage gap (that is, people eligible for Medicaid, ACA Marketplace plans, or employer-sponsored coverage); we considered these people to not have uncovered costs for PrEP.

Among people having uncovered costs, some could receive PrEP medication (not including clinical visit or laboratory costs) at no cost through a manufacturer’s medication assistance program (for example, Gilead’s Advancing Access program), which is offered to uninsured US residents with a household income less than 500 percent of the federal poverty level.^20^ We classified people without PrEP insurance coverage and with a household income less than 500 percent of poverty as having uncovered costs for PrEP clinical care, as these people would be eligible for a manufacturer’s medication assistance program. We classified people without PrEP insurance coverage and with a household income of at least 500 percent of poverty as having uncovered costs for PrEP medication and clinical care (that is, all PrEP-related costs), as these people would not be eligible for such a program.

### Population Inputs

Data from the National HIV Surveillance System and the National Health and Nutrition Examination Survey and census data were used to estimate the number of people age eighteen or older with indications for PrEP in 2018. These estimates were stratified by the following HIV transmission risk groups: gay, bisexual, and other men who have sex with men (collectively referred to as MSM); heterosexual men and women; and people who inject drugs, as previously described.^21,22^

We used data from the 2017–19 National Survey of Family Growth^23^ and the 2019 National Survey on Drug Use and Health^24^ to estimate the percentages of people by federal poverty level and insurance coverage for the above HIV transmission risk groups. We then assumed that those percentages also applied to the subset of people with PrEP indications within each risk group. We categorized poverty by the percentage of people with household incomes either less than 500 percent or at least 500 percent of poverty. The National Survey of Family Growth included a calculated variable for federal poverty level. For people who inject drugs, the National Survey on Drug Use and Health did not include a corresponding calculated variable, so we estimated federal poverty level using the Census Bureau’s weighted average poverty thresholds for 2020.^25^ We categorized insurance types as private, public, and uninsured (see appendix B for further detail on insurance categorization).^18^ We used 2021 estimates from the Henry J. Kaiser Family Foundation to characterize uninsured people who were ineligible for insurance because of immigration status or being in the Medicaid coverage gap.^19^ We assumed that those estimates applied equally across transmission risk groups and without regard to PrEP indications.

### Cost Inputs

We estimated the costs of PrEP medication and clinical care from multiple sources. We estimated the net Medicaid price for a thirty-day supply of brand-name Truvada and Descovy (the two oral PrEP medications in use in 2018) as a proxy for 340B drug ceiling prices,^26^ and we used the National Average Drug Acquisition Cost database^27^ to estimate the price for a thirty-day supply of generic TDF/FTC (see appendix C for further detail on the estimation of PrEP medication costs).^18^ The cost of clinical visits for the first year was based on national estimates from the Centers for Medicare and Medicaid Services (CMS) 2021 Medicare Physician Fee Schedule for specific Current Procedural Terminology billing codes.^28^ The cost of lab tests for routine PrEP assessment and clinical monitoring for one year was estimated using the CMS 2021 Clinical Laboratory Fee Schedule.^29^ Cost inputs for PrEP clinical visits and lab testing were applied uniformly across groups.

Annual laboratory costs were calculated by transmission risk group, as clinical monitoring recommendations in the 2021 PrEP clinical practice guideline vary by group^2^ (see appendix D for further detail on the estimation of annual laboratory costs).^18^

Using the estimates of people with PrEP indications by transmission risk group with uncovered costs for PrEP medication and clinical care and for PrEP care only, we estimated the total annual uncovered costs for US adults with PrEP indications.

### Sensitivity Analyses

We conducted sensitivity analyses evaluating variation in three key inputs, including the estimated percentage of people with income less than 500 percent of poverty by insurance status, estimated PrEP medication costs, and estimated costs if no medication assistance program were available (see appendix E for further detail on sensitivity analyses methods).^18^

### Limitations

This analysis was subject to several limitations. First, multiple population inputs (for example, insurance status or being in the Medicaid coverage gap) were not specifically available stratified by transmission risk group or by people with PrEP indications. In those instances, we assumed that inputs applied equally across groups.

Second, we estimated that 1 percent of insured people had PrEP-related claims denied; however, the frequency at which this occurs is not well described in the literature.

Third, 340B ceiling prices are proprietary, and there is no consensus method for their estimation. Given this, we estimated net Medicaid prices as a proxy and performed a sensitivity analysis using another cost source to evaluate uncertainty in 340B ceiling prices (see appendixes C and E for detail on PrEP medication cost estimates and sensitivity analyses).^18^ In addition, National Average Drug Acquisition Cost prices used to estimate the cost for generic TDF/FTC do not include discounts or rebates paid to pharmacies, health plans, or pharmacy benefit managers.

Fourth, our analytic framework did not account for cost sharing imposed by private insurance plans exempted from PrEP cost-sharing requirements under the ACA (for example, grandfathered plans and short-term limited-duration plans), for which there is minimal information available regarding their PrEP coverage policies. The analysis did not account for public insurance plans that impose cost sharing for PrEP, which may include Medicare (approximately 3 percent of PrEP prescriptions are paid by Medicare, according to 2020 IQVIA PrEP prescription data) and some traditional Medicaid programs.^30,31^ This analysis did does not account for the Department of Health and Human Services Ready, Set, PrEP program or state and jurisdictional PrEP assistance programs.^15,16^ The total annual uncovered costs reported here may overestimate the true value, as additional people may be eligible for these PrEP assistance programs.

Finally, long-acting injectable cabotegravir, which received Food and Drug Administration approval for PrEP use in December 2021,^32^ was not included in our framework. Few people are receiving this medication for PrEP, and there is uncertainty regarding possible medication assistance programs, insurance coverage, and uptake for it. Early reports suggest that the annual cost will be similar to that for brand-name oral PrEP,^33^ and this analysis can be updated to include cabotegravir-related inputs in the future.

## Study Results

We estimated that in the 2018 population of US adults with PrEP indications, 71 percent of MSM had private insurance, 15 percent had public insurance, and 14 percent were uninsured (exhibit 1). Among heterosexual women and men, 56 percent and 57 percent had private insurance, 28 percent and 19 percent had public insurance, and 15 percent and 24 percent were uninsured, respectively. Among people who inject drugs, 18 percent had private insurance, 60 percent had public insurance, and 23 percent were uninsured. The percentage of adults with PrEP indications who had incomes below 500 percent of poverty ranged from 68 percent to 100 percent, varying by transmission risk group and insurance type. Thus, summing insurance category totals over the overall population of US adults with PrEP indications, 64 percent had private insurance, 21 percent had public insurance, and 16 percent were uninsured (these percentages sum to more than 100 percent because of rounding).

Exhibits 2 and 3 describe the estimated number of people with PrEP indications who had uncovered costs for PrEP in 2018, after accounting for existing insurance coverage and manufacturer assistance. An estimated 32,350 MSM (4 percent of MSM with PrEP indications) had uncovered costs for PrEP, 25,020 of whom were uninsured. An estimated 7,600 heterosexual women (4 percent of heterosexual women with PrEP indications) had uncovered costs for PrEP, 6,010 of whom were uninsured. An estimated 5,070 heterosexual men (6 percent of heterosexual men with PrEP indications) had uncovered costs for PrEP, 4,410 of whom were uninsured. An estimated 4,840 people who inject drugs (6 percent of people who inject drugs with PrEP indications) had uncovered costs for PrEP, 4,170 of whom were uninsured. Among uninsured people with PrEP indications, an estimated 24,520 (13 percent) were ineligible for insurance coverage because of immigration status, and 15,090 (8 percent) were in the Medicaid coverage gap. In total, an estimated 49,860 people with PrEP indications had uncovered costs for PrEP in 2018.

The estimated total uncovered costs for PrEP medication and clinical care are described in exhibit 4. The cost of PrEP medication, using a weighted average of prices for generic TDF/FTC, Truvada, and Descovy, was $4,196 per person in 2018. The annual cost of PrEP clinical care was $671 for clinical visits, and laboratory test costs ranged from $478 for heterosexual men and women to $1,458 for MSM. The total annual cost of PrEP medication and clinical care ranged from $5,345 for heterosexual men and women to $6,325 for MSM; for PrEP clinical care alone, this ranged from $1,149 for heterosexual men and women to $2,129 for MSM.

Among the 49,860 total people with uncovered costs for PrEP in 2018, an estimated 3,160 (6 percent) had uncovered costs for both PrEP medication and clinical care, at an estimated annual cost of approximately $18.9 million (exhibit 4). Of these people, 2,020 were MSM, 460 were heterosexual women, and 680 were heterosexual men; no people who inject drugs had uncovered costs for both PrEP medication and clinical care. The remaining 46,700 (94 percent) had uncovered costs for PrEP clinical care only (that is, these people were eligible for manufacturers’ medication assistance programs), at an estimated annual cost of approximately $83.5 million. Of these 46,700 people, 30,330 were MSM, 7,140 were heterosexual women, 4,390 were heterosexual men, and 4,840 were people who inject drugs. The overall estimated annual uncovered cost for PrEP in 2018 was approximately $102.4 million, of which $13.3 million was for PrEP medication, $33.5 million was for clinical visits, and $55.7 million was for laboratory testing.

In the sensitivity analysis, using varied inputs for the estimated percentage of people with income less than 500 percent of poverty, the total number of people with uncovered costs remained unchanged, and estimated total annual uncovered costs ranged from $99.4 million to $105.7 million (see appendix exhibits 2A–2E).^18^ Using a secondary source for PrEP medication prices, the annual cost for PrEP medication increased to $12,507 per person, raising estimated total annual uncovered costs to $128.6 million (see appendix exhibit 3).^18^ In the scenario without a medication assistance program, estimated total annual uncovered costs increased to $298.3 million (see appendix exhibit 4),^18^ as all people’s costs would be uncovered for both PrEP medication and clinical care.

## Discussion

Among the 1.2 million US adults with PrEP indications in 2018, we found that nearly 50,000 people had uncovered costs for PrEP, with more than 90 percent having uncovered costs for PrEP clinical care only because they already qualified for PrEP medication payment assistance. In total, fewer than 4 percent of US adults with PrEP indications had uncovered costs for PrEP clinical care only, and fewer than 1 percent had uncovered costs for both PrEP medication and clinical care. Total annual uncovered costs were approximately $102 million in 2018.

Overall, an estimated 86 percent of MSM, 85 percent of heterosexual women, 76 percent of heterosexual men, and 77 percent of people who inject drugs had public or private insurance to cover PrEP medications and clinical care in 2018. Among the 16 percent of adults with PrEP indications who were uninsured in 2018, our analysis suggests that 79 percent were eligible by income and immigration status (those listed in the “Documented, insurance eligible” rows of exhibits 2 and 3) for health insurance coverage through ACA Marketplaces (potentially including eligibility for tax credits), Medicaid or other public insurance, or employer-sponsored plans.^19^ Although affordability is frequently cited by uninsured US adults as the most common barrier to coverage, more than 50 percent of uninsured people may be eligible for subsidized coverage through Medicaid or ACA Marketplaces.^19,34,35^

In addition, policies that increase access to health insurance, such as Medicaid expansion, can improve access to PrEP. This may be especially impactful for the southern US, where 97 percent of the 2.2 million adults who fall into the Medicaid coverage gap live and which has the lowest PrEP-to-need ratio (the number of PrEP users divided by new HIV diagnoses) in the US.^36,37^

Compared with the prior 2015 analysis by Smith and colleagues,^11^ the estimated number of people with uncovered PrEP costs decreased from 93,630 in 2015 to 49,860 in 2018, a 47 percent decrease. Estimates for the total annual uncovered costs for PrEP also decreased by more than 50 percent, going from approximately $208 million to $102 million in that time frame.

There are a few notable reasons for the decreases. First, the number of people with uncovered costs for PrEP medication and clinical care declined. For each transmission risk group, a smaller percentage of people were uninsured in 2018 than in 2015, with the largest insurance gains being private insurance for MSM and heterosexual men and women and public insurance for people who inject drugs. These insurance coverage gains can be attributed to continued implementation of the ACA and additional states expanding Medicaid.^38^ Second, a higher proportion of uninsured people with PrEP indications had incomes below 500 percent of poverty in 2018 than in 2015. This has contributed to reduced financial costs for a payer of last resort, such as a national PrEP assistance program, as those people are already eligible for medication payment assistance. Because fewer people had uncovered costs for PrEP medication, PrEP clinical care costs accounted for most of the uncovered costs. Third, the introduction of generic TDF/FTC greatly reduced the estimated annual cost of PrEP medication. Whereas the 2015 analysis estimated the cost of Truvada (the only available PrEP medication at the time) to be $10,770 per year, the current analysis estimated the cost at $4,196 in 2018, using a weighted average of Truvada, Descovy, and generic TDF/FTC. As 99 percent of privately and publicly insured people were considered to have adequate PrEP coverage in both the prior and current analyses, the US Preventive Services Task Force’s 2019 grade A recommendation for oral PrEP—another key development in reducing the out-of-pocket expense for PrEP—did not contribute to differences in our estimates. It did increase confidence in our estimates, as Medicaid expansion coverage and all nonexempt private insurance plans may not impose cost sharing for PrEP medications or care as of January 1, 2021. However, uptake of these new PrEP coverage provisions has been incomplete.^39^

Although estimated uncovered costs have decreased over time, an amount of more than $100 million annually would still be significant for a national PrEP assistance program to address. For context, total domestic HIV spending in the US was approximately $28 billion for fiscal year 2019.^40^ More than 75 percent of this was spent on HIV care and treatment services; HIV prevention accounted for approximately $900 million. Notably, our results likely overestimate real-world costs, as the model assumed that everyone with PrEP indications who would have uncovered costs would be prescribed PrEP. The most recent estimate of PrEP coverage, or the percentage of people prescribed PrEP among all of those with PrEP indications, in the US was 30 percent for 2021.^4^ Given current trends in PrEP coverage and considering the EHE initiative’s PrEP coverage goal of 50 percent by 2025, real-world PrEP coverage rates will likely be well below 100 percent for the next few years.

Significant racial, ethnic, and geographic inequities in PrEP use persist throughout the US.^1,6,36^ Overcoming barriers to PrEP, among them being financial barriers, remains essential to increasing PrEP use equitably for communities disproportionately affected by HIV infection and to achieving the goals of the EHE initiative. Whether through reducing the number of uninsured people or extending the reach of financial assistance programs, expanding coverage for PrEP medication and clinical care can positively affect PrEP scale-up.

A few proposals to establish and fund a national PrEP program have recently emerged. These include a new program proposed in the FY 2023 president’s budget, two congressional bills, and a policy proposal from Johns Hopkins University.^41–44^ Although the baseline year for this analysis was 2018, we believe that our estimates can inform the ongoing policy discussion regarding a national PrEP program. Our cost estimates likely closely reflect present-day estimates, as the population of US adults with indications for PrEP—the primary lagging input in this analysis—changes minimally year to year. For example, the estimate decreased less than 0.5 percent from 2017 to 2018.^22^ In addition, the analysis was structured to reflect changes to the PrEP landscape through 2021, including the introduction of generic PrEP, the 2021 PrEP clinical practice guideline release, and other updated cost inputs.^2,17^

## Conclusion

PrEP is a powerful HIV prevention tool and will continue to play an essential role in achieving the goals of the EHE initiative. This analysis found that the number of people with uncovered costs for PrEP medication and clinical care is less than 5 percent among US adults with PrEP indications, but the sum of those uncovered costs is significant. Understanding how the number of people with PrEP-related uncovered costs and the corresponding total costs have changed over time is an important consideration for policy makers when evaluating how to overcome financial barriers to PrEP use and decrease new HIV infections in the US.

## Figures and Tables

**EXHIBIT 1:** Estimates of insurance status and preexposure prophylaxis (PrEP) costs, by HIV transmission risk group, United States, 2018

Population inputs	Total	MSM	HET women	HET men	PWID
No. of adults with PrEP indications	1,216,210	851,240	188,020	86,660	87,530
Insurance status (%)					
Private	63.6^a^	70.9	56.4	56.7	17.6
Public	20.6^a^	15.1	28.4	19.1	59.7
Uninsured	15.5^a^	14.0	15.2	24.2	22.7
Among the uninsured (%)					
In the coverage gap	8	8	8	8	8
Ineligible for coverage due to immigration status	13	13	13	13	13
Income <500% of FPL (%)					
Privately insured adults	71.5^a^	68.1	75.2	71.9	99.0
Publicly insured adults	93.2^a^	92.7	97.1	86.3	100.0
Uninsured adults	98.5^a^	100.0	97.0	88.1	100.0
Cost inputs^b^	Unit cost	MSM	HET women	HET men	PWID
Clinician visit costs ($)					
Initial assessment visit (CPT 99204)	169.93	169.93	169.93	169.93	169.93
Initial prescription visit (CPT 99213)	92.47	92.47	92.47	92.47	92.47
Quarterly follow-up visit (CPT 99213)	92.47	92.47	92.47	92.47	92.47
Annual follow-up visit (CPT 99214)	131.20	131.20	131.20	131.20	131.20
Visits for first year, total	671.01	671.01	671.01	671.01	671.01
Clinical lab costs^c^ ($)					
4th generation HIV antigen and antibody test (CPT 87389)	24.08	120.40	120.40	120.40	120.40
HIV RNA test (CPT 87535)	35.09	140.36	140.36	140.36	140.36
Basic metabolic panel (CPT 80048)	8.46	16.92	16.92	16.92	16.92
Hepatitis B serology (HBsAg, HBsAb, total HBcAb) (CPT 87340, 86706, 86704)	33.12	33.12	33.12	33.12	33.12
Hepatitis C serology (CPT 86803)	14.27	28.54	—^d^	—^d^	28.54
Syphilis serology (CPT 86780)	13.24	66.20	26.48	26.48	26.48
NAAT GC/CT (per site sampled, 1 for HET M + W, 3 for MSM) (CPT 87491, 87591)	70.18	1,052.70	140.36	140.36	140.36
Medication costs, 30-day supply ($)					
Truvada 340B price	409.66	409.66	409.66	409.66	409.66
Generic TDF/FTC 340B price	26.04	26.04	26.04	26.04	26.04
Descovy 340B price	518.09	518.09	518.09	518.09	518.09
Weighted average of PrEP medications^e^	349.70	349.70	349.70	349.70	349.70

**Sources** Adults with PrEP indications: Centers for Disease Control and Prevention (CDC) (note 22 in text). Insurance status and income levels: authors’ calculations based on the 2017–19 National Survey of Family Growth (NSFG) and the 2019 National Survey on Drug Use and Health (NSDUH) (notes 23 and 24 in text). Uninsured, in the coverage gap and ineligible for coverage due to immigration status: Henry J. Kaiser Family Foundation (note 19 in text). Clinician visit costs: Centers for Medicare and Medicaid Services (CMS) 2021 Physician Fee Schedule (note 28 in text). Clinical lab costs: CMS 2021 Clinical Laboratory Fee Schedule files (note 29 in text). Medication costs: authors’ calculations based on 2021 Federal Supply Schedule and National Average Drug Acquisition Cost data (notes 26 and 27 in text). **Notes** MSM is men who have sex with men. HET is heterosexual. PWID is people who inject drugs. FPL is federal poverty level. CPT is Current Procedural Terminology. HBsAg is hepatitis B surface antigen. HBsAb is hepatitis B surface antibody. HBcAb is hepatitis B core antibody. NAAT is nucleic acid amplification test. GC is *Neisseria gonorrhoeae*. CT is *Chlamydia trachomitis*. M + W is men plus women. TDF is tenofovir disoproxil fumarate. FTC is emtricitabine.

a Value represents the weighted average of this measure from each transmission risk group (across the row), as it cannot be directly calculated from the NSFG or the NSDUH for the “total” group.

b Cost inputs represent unit costs unless otherwise noted.

c Laboratory costs displayed by transmission risk group represent the cumulative annual cost for each lab test based on the frequency with which the test is indicated for that group.

d Not indicated for heterosexual men and women.

e Medication costs calculated as weighted average of estimated prices for Truvada, Descovy, and generic TDF/FTC, based on the distribution of PrEP prescriptions in the IQVIA database for the fourth quarter of 2020.

**EXHIBIT 2:** Estimated number of adults in different cost coverage categories for preexposure prophylaxis (PrEP) medication and clinical care, by insurance status and HIV transmission risk group, United States, 2018

Cost coverage categories	MSM	HET women	HET men	PWID
**PRIVATE INSURANCE**				
Adults with PrEP indications	603,530	106,040	49,140	15,410
PrEP payment support unavailable				
Insurance denies payment	6,040	1,060	490	150
PrEP payment support available				
Insurance coverage for PrEP	597,490	104,980	48,650	15,260
People with uncovered costs	6,040	1,060	490	150
For PrEP clinical care^a^	4,110	800	350	150
For PrEP medication and clinical care^b^	1,930	260	140	0
**PUBLIC INSURANCE**				
Adults with PrEP indications	128,540	53,400	16,550	52,260
PrEP payment support unavailable				
Insurance denies payment	1,290	530	170	520
PrEP payment support available				
Insurance coverage for PrEP	127,250	52,870	16,380	51,740
People with uncovered costs	1,290	530	170	520
For PrEP clinical care^a^	1,200	510	150	520
For PrEP medication and clinical care^b^	90	20	20	0
**UNINSURED**				
Adults with PrEP indications	119,170	28,580	20,970	19,870
PrEP payment support unavailable				
Ineligible for coverage because of immigration status	15,490	3,720	2,730	2,580
In coverage gap	9,530	2,290	1,680	1,590
PrEP payment support available				
Documented, insurance eligible^c^	94,140	22,580	16,570	15,700
People with uncovered costs	25,020	6,010	4,410	4,170
For PrEP clinical care^a^	25,020	5,830	3,890	4,170
For PrEP medication and clinical care^b^	0	180	520	0

**Source** Authors’ calculations based on data from exhibit 1. **Notes** All estimates are rounded; totals might not sum secondary to rounding. MSM is men who have sex with men. HET is heterosexual. PWID is people who inject drugs.

a Eligible for manufacturer medication assistance program, determined by income below 500% of the federal poverty level.

b Not eligible for manufacturer medication assistance program, determined by income of at least 500% of the federal poverty level.

c Eligible for Medicaid, Affordable Care Act tax credits, or other insurance.

**EXHIBIT 3:** Estimated number of adults in different cost coverage categories for preexposure prophylaxis (PrEP) medication and clinical care, by HIV transmission risk group, United States, 2018

Cost coverage categories	MSM^a^	HET women^a^	HET men^a^	PWID^a^	Row total
Adults with PrEP indications	851,240	188,020	86,660	87,540	1,216,210
PrEP payment support unavailable Insurance denies payment	7,330	1,590	660	670	10,250
Ineligible for coverage due to immigration status	15,490	3,720	2,730	2,580	24,520
In coverage gap	9,530	2,290	1,680	1,590	15,090
PrEP payment support available					
Insurance coverage for PrEP	724,740	157,850	65,030	67,000	1,014,620
Documented, insurance eligible^b^	94,140	22,580	16,570	15,700	148,990
People with uncovered costs	32,350	7,600	5,070	4,840	49,860
For PrEP clinical care^c^	30,330	7,140	4,390	4,840	46,700
For PrEP medication and clinical care^d^	2,020	460	680	0	3,160

**Source** Authors’ calculations based on data from exhibit 2. **Notes** MSM is men who have sex with men. HET is heterosexual. PWID is people who inject drugs.

a Total number of people across insurance categories in exhibit 2 for the corresponding transmission risk group.

b Eligible for Medicaid, Affordable Care Act tax credits, or other insurance.

c Eligible for manufacturer medication assistance program, determined by income below 500% of the federal poverty level.

d Not eligible for manufacturer medication assistance program, determined by income of at least 500% of the federal poverty level.

**EXHIBIT 4:** Estimated annual cost for preexposure prophylaxis (PrEP) medication and clinical care for adults with uncovered costs, by HIV transmission risk group, United States, 2018

	MSM	HET women	HET men	PWID	Total
**MEDICATION, CLINICAL VISITS, LABS**					
No. with uncovered costs	2,020	460	680	0	3,160
Annual cost of PrEP medication and clinical care, per person ($)					
PrEP medication^a^	4,196	4,196	4,196	4,196	13,260,450
Clinical visits^b^	671	671	671	671	2,120,360
Lab costs^c^	1,458	478	478	506	3,490,080
Total	6,325	5,345	5,345	5,373	18,869,800
**CLINICAL VISITS AND LABS ONLY** ^d^					
No. with uncovered costs	30,330	7,140	4,390	4,840	46,700
Annual cost of PrEP clinical care, per person ($)					
Clinical visits^b^	671	671	671	671	31,335,700
Lab costs^c^	1,458	478	478	506	52,181,520
Total	2,129	1,149	1,149	1,177	83,517,220
**COMBINED COST FOR ALL WITH UNCOVERED COSTS**					
Grand total ($)	—^e^	—^e^	—^e^	—^e^	102,387,020

**Source** Authors’ calculations based on data from exhibits 1–3. **Notes** All estimates are rounded; totals might not sum because of rounding. MSM is men who have sex with men. HET is heterosexual. PWID is people who inject drugs.

a Calculated as weighted average of estimated prices for Truvada, Descovy, and generic tenofovir disoproxil fumarate/emtricitabine, based on the distribution of PrEP prescriptions in the IQVIA database for the fourth quarter of 2020.

b Source: Centers for Medicare and Medicaid Services (CMS) 2021 Physician Fee Schedule (note 28 in text).

c Source: CMS 2021 Clinical Laboratory Fee Schedule (note 29 in text).

d Eligible for manufacturers’ medication assistance programs.

e Not applicable.
